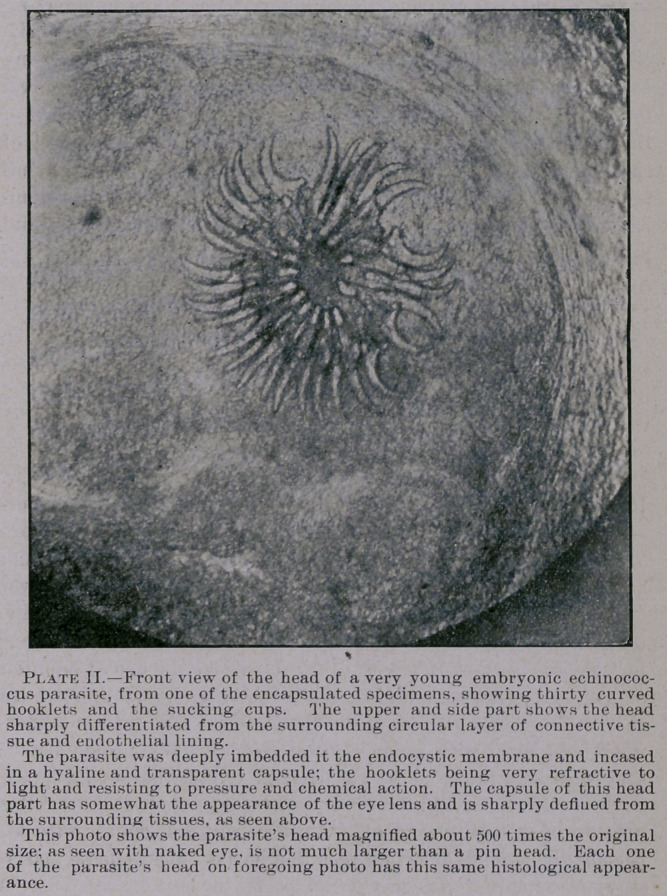# Some Further Observations on the Echinococcus Disease, or Bladder Tapeworm

**Published:** 1900-02

**Authors:** Rudolph Menger

**Affiliations:** San Antonio, Texas


### TEXAS MEDICAL JOURNAL.

ESTABLISHED JULY, 1885.

PUBLISHED MONTHLY.—SUBSCRIPTION $1.00 A YEAR.

VOL. XV. AUSTIN, FEBRUARY, 1900. No. 8.

### Original Contributions.

### Some Further Observations on the Echinococcus Disease, or Bladder Tapeworm.

BY RUDOLPH MENGER, M. D., SAN ANTONIO, TEXAS.

PART II.

*Editors Texas Medical Journal:*

In order to make the first report on the subject heading these lines more complete, and in order to advance the literature, and especially the histology of these—in Texas at least—it seems, not much known parasites as to their true nature, I respectfully submit further data. And, in order to give a short account of the more common type of tapeworms found in man and animals, I include herewith the following clipping from the pen of some other writer:

"There are many species of that heartless robber, the tapeworm, but the two most common as human parasites are the beef tapeworm and the pork tapeworm, so called from the animals they inhabit. The beef tapeworm or tænia saginata, varies from ten to thirty-five feet in length, and is more than an inch wide at the widest part. The body consists of a head followed by a chain of segments. The head, one-fifth of an inch wide, possesses four muscular pouches or suckers, by means of which it clings to the wall of an intestine. The life history of this tapeworm is very strange. When the eggs escape from the body they possess a hard shell and can live for some time. If one of these eggs is taken in with the grass or fodder eaten by cattle, the egg will develop. If not, it dies. Thus there is only one chance in ten thousand that a tapeworm egg develops. When the egg comes into a cow's stomach the hard shell is destroyed and the little worm contained is set free. It immediately bores through the wall of the stomach or intestine and finds its way into the muscles or some organ of the animal. Here it remains stationary and grows larger until it becomes one-third of an inch long. Finally it stops growing and forms a shell around itself. In this stage it is called a bladder worm, because its body looks like a sack with the head inclosed. When these bladder worms are present in large numbers the meet is called 'measley beef.' If the beef which contains the bladder worm is eaten by man the worm will be released by the digestion of the meat and will be set free in the stomach or small intestine. By means of its four suckers it fastens itself to the wall of the intestine and begins to grow. It grows with wonderful rapidity, forming ten or more segments and increasing its length three inches daily.

"When we examine the structure of a tapeworm, we find that it has no trace of a digestive system. It is not hard to see the reason why. The tapeworm simply waits until the food is digested by the stomach and small intestine and then absorbs it through its skin. Man not only furnishes it lodging and food, but also digests the food. The tapeworm is dangerous for this reason: The unfortunate simply starves because his food is stolen. In addition, the presence of the worm causes irritation in the intestines. The beef tapeworm is becoming more numerous. In France, from 1861 to 1865 there was only one man infected out of 5000. From 1886 to 1899, there were seventy-five in 5000. There is probably a good increase in the United States also, which can be traced to this reason: Rare done beef is increasing in favor, and consequently the beef tapeworm is becoming more common. The only way that you can be sure of not being infected is to take your steak well done. The danger from these parasites is so great that every packing house has the meat examined, before it is used, by a government official belonging to the bureau of animal industry. The pork tapeworm, tænia colium, is rare in the United States, because we take our pork well done. It differs from the beef tapeworm in having a double ring of hooks in addition to the four suckers. Its bladder worm stage forms in hogs, and therefore infection comes from eating pork. It is more dangerous than the beef tapeworm, because its eggs may develop in a man directly without going to another animal. In such a case the worm leaving the egg bores through the walls of the stomach of man just as it would through that of a pig, and thus causes inflammation and serious trouble by penetrating important organs. Well done meats prevent tapeworms."

Now, regarding the *echinococcus parasite* or *bladder tapeworm embryo* causing cystic disease in the prairie rabbit, an account of which has been given in a previous edition of the JOURNAL, I wish to add the following observations, which, I believe, will give a little better insight into the pathology of the bladder-tapeworm family causing cystic disease in man and animals. This investigation will show, that after the development of the premature or embryonic parasite of the echinococcus tapeworm from the ingested ova of the mature tænia, up to the migration, encystment and sprouting of new embryonic colonies inside the cyst membrane in different parts or organs of the human and animal system, that all of these parasites in their most primitive stadia already show quite a well-developed head with characteristic suckers and hooklets (Plates I and II), and that these, after further development, free themselves from the endocystic membrane, although still adherent to its lining; and ultimately, after still further development, some of the more mature of these embryonic parasites isolate and free themselves entirely from the other more premature crop and can then be found in a free state either near the base of the endo-cystic membrane or inside the cyst fluid.

The illustrations in this second paper were made from microscopic specimens obtained lately from a cystic diseased prairie rabbit. The cysts were rather small in this instance, but numerous and conglomerated, resembling closely a bunch of the large California grapes, and were situated in the thoracic cavity near the base of the lungs, between the œsophagus and bifurcation of the trachea, apparently adherent to the pleura. A whole handful of these cysts, or so-called hydradites, were removed, and they were not larger than a small marble, of oval shape, and transparent. From the base of these cystic conglomerations a particle of the endocystic membrane was removed for microscopic examination. (See micro-photo I, this article.) In its crude or microscopic appearance several globular bodies could be seen with a strong loup, and these, on examination with the microscope, revealed at once their true nature. After preparing this piece of membrane with acetic acid and glycerine and staining afterward, all the parasites showed up beautifully,—succeeded also in preparing the fine photo copies seen in the JOURNAL.

There could be counted about twenty-five of these parasites in a space not larger than about one-fourth of an inch. The parasites were in different states of development, but all still encased in a separate lining of the basement membrane. On pressure between two slide glasses the capsule surrounding the parasite would crack and expose the embryonic parasite more fully. It seems, after the head of the parasite (Plate II) which, with its thirty curved and sharp pointed hooks, is very resisting to chemical action and pressure, etc., has been imbedded in the tissues, a severe cell and tissue proliferation is started up which gradually entirely encases the parasite.

This process is beautifully illustrated in the above micro-photographs.

Plate I. shows the head of one of these parasites magnified about 500 times. (Originally, as seen with the naked eye, they are, including the encasing capsule, not larger than a pin's head, and nearly all are situated at the edge or end part of the peculiarly fimbricated endocystic membrane.)

It seems to me, that the more fully developed of these embryonic tænia species are the ones that are liable to perforate the cyst membrane and migrate into other organs and there develop into the large echinococcus cyst and sprout hundreds of young offspring within and at the base of the cyst sack. Undoubtedly, though, the direct primary cause of the disease in man and animals is the matured ova of this, the smallest tapeworm species known to science, which is harbored, as stated fully in the previous paper, by the dog, wolf, fox, etc., and accidentally transmitted through the food or drinking water upon man or animal. And while this is the accepted theory, the question arises: "Can the echinococcus parasite or hydradite disease in man, as far as Texas is concerned, also be contracted from the same species of embryo cocci of rabbit, when such infected rabbit meat is accidentally eaten?" Experimental tests, of course, only could determine this. So far, though, without positive and direct proof, it is only problematic.

In conclusion, I may state that the publication of the first paper in this journal has caused quite an interest—according to letters received, including the one of Dr. F. Herff, and one from Dr. R. H. Bibb, of Mexico. Dr. Bibb wrote that he had also removed echinococcus tumors in man, the parasites being revealed by the microscope. Hydradites and cysts also have been removed by several of the elder physicians of San Antonio. Dr. Julius Braunnagel of this city, I may state in connection with these publications, has also taken a deep interest in this matter and he submitted a number of prepared slides to a close examination in his private and up-to-date bacteriological laboratory.

## Figures and Tables

**Plate. I. f1:**
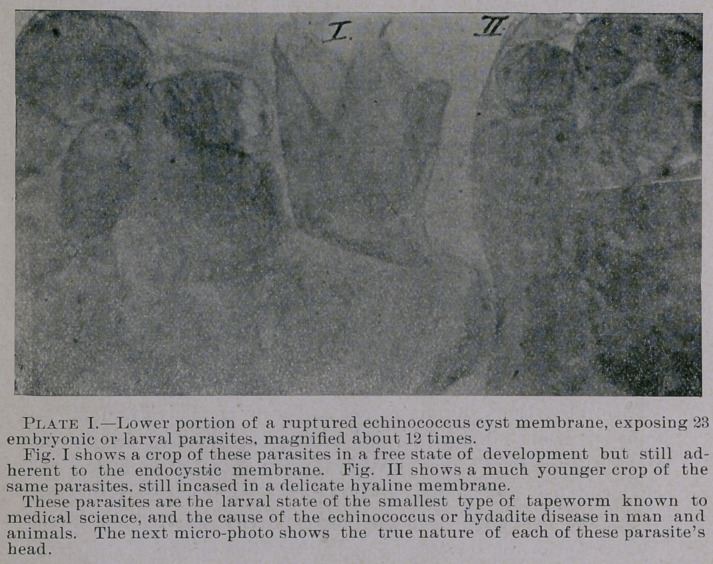


**Plate. II. f2:**